# Frustrations of a Longtime Global Issues Activist: Comment on "Ensuring Global Health Equity in a Post-pandemic Economy"

**DOI:** 10.34172/ijhpm.2023.8242

**Published:** 2023-10-23

**Authors:** Claudio Schuftan

**Affiliations:** People’s Health Movement, Ho Chi Minh City, Vietnam.

**Keywords:** Flawed Global Governance, Cooptation of UN Bodies, Defending Multilateralism, Resisting Multistakeholderism, Needed Engagements

## Abstract

Labonté’s first commentary^1^ concluded with what I wholeheartedly agree, namely that "we need an activist public health movement to ensure there is sufficient political will to adopt them." In their follow-up commentary, Moers and colleagues^2^ looked at things from a slightly different angle saying that to achieve equity will need radical changes in economic thinking and policies; they added that advocates needed to be strategic about framing and use hope-based communication and develop attractive and convincing narratives: "By doing so, hopefully we can bring these messages across to larger groups of people." Well, I think that, together with many others, I have been strategic and radical, but only to accumulate a large bag of disappointments and broken hopes in trying to ‘bring the message across.’ But I come back to memories of so many defeats that I, with others, have lived through. Here, I describe my frustrations but explain why I do not give up hope.

## Can We Work Towards Building a People’s Governance Grounded in Multilateralism and Human Rights?

*It just takes courage to stand up for the things we do at this moment of history*.

 Frustrations can cause us to brood, but they also make us rethink. It is perhaps pertinent here to recall a few of the negotiations I was involved in that justify my frustration: negotiation of Food and Agriculture Organization’s (FAO’s) Voluntary Guidelines on the Right to Food and those on Food Systems, reforming FAO’s Committee on Food Security, the introduction of a coordinated COVID-19 and current food crisis action agenda at the same Committee on Food Security, the COVID-19 waiver at the World Trade Organization allowing the transfer of vaccine technology, the United Nations’ (UN’s) Food Systems Summit that ended up being a showcase of corporate capture and conflicts of interest, the Binding Treaty on Transnational Corporations and Human Rights being painfully negotiated for nine+ years at the Human Rights Council, World Health Organization’s (WHO’s) Framework of Engagement with Non-State Actors and other WHO resolutions, the Scaling Up Nutrition Initiative, UN Nutrition rising from the demise of the UN’s Standing Committee on Nutrition, WHO’s COVID-19 Vaccines Global Access Initiative (COVAX), 26 Conferences of the Parties on Climate Change since the Rio Earth Summit in 1992… And the list could go on… The common denominator here is that the unambiguous position of public interest organizations, literally representing millions, did not fare too well in all of these thus the frustration I here ventilate.

 In this commentary I want to zero-in on the challenges social movements actors have chronically faced in relation to having so unsuccessfully tackled and continue to tackle global governance issues. Am I and these actors fooling ourselves that ‘things are going to eventually be alright?’ or Does everybody involved need to work in a totally different way given that the private sector and countries rendered rich have pushed our backs totally against the wall on these issues and fora? My hope is that smart young people pick up the challenges I depict below.

## Bringing the Relevant Issues to Mind (Needed Elements for a Cool-Headed Analysis)

 Public interest civil society organizations (PICSOs) and social and indigenous movements forever seek meaningful participation in global fora in an effort to influence and strengthen — beyond voice — the decisions that can lead to lasting, legally binding changes. Unfortunately, too many times their pleas are ultimately ignored. But, they keep trying despite all odds: …“*I participate. You participate. He/she participates. We participate; but… They decide*” (chalkboard in La Paz, Bolivia).

 Risking being brief to the point of offering only a caricature, I here distill my experience on the most relevant issues: (I have written in more detail explaining and backing up each of these bullet points. If interested, go to https://claudioschuftan.com/133-frustrations-of-a-lifelong-global-issues-activist/)

- The UN system is fatally flawed as the basis for multilateral/sectoral agreements and needs wholesale reform; but this reform can and will only ‘come from below.’
- As said, PICSOs have insufficient power to influence UN-related negotiations yet are often better informed/resourced than lower- and middle-income country delegations who vote for relevant resolutions.
- Opportunities are given to PICSOs simply to give the illusion of genuine consultation/inclusivity.
- Final decisions that PICSOs try to influence too often clash with the call for consensus-arrived resolutions by UN bodies and member states. It remains to be proven though that such a consensus is reached by genuine choice or by pressure reflecting an international system captured by the powerful influence of countries rendered rich.
- Michael Fakhri, the UN Special Rapporteur for the Right to Food reminded us that “PICSOs coming to the table to discuss better, global solutions’ is not as simple as it sounds, especially if the table is already set, the seating plan non-negotiable and the menu highly limited. …And what if the real conversation is actually happening at a different table?”^3^
- Consensus using softened language is usually hammered out at the wee-wee hours of the night before the deadline a resolution must be passed — only to make PICSOs bitterly complain.
- Business interest non-governmental organizations are significantly more powerful in UN-related negotiations both directly and indirectly as members of multistakeholder platforms and public-private partnerships that lobby at country level and at international UN agencies (To little avail, civil society actors incessantly and forcefully denounce and dispute this).
- The global political economy continues to concentrate resources in the hands of private actors so that the international rules-based system of global governance currently enables, rather than resists their influence.
- The drivers of global governance have access to enormous and growing resources so that those rendered rich will find more and more ways to resist regulations that hamper their interests.
- A global conscience raising effort is needed to frame and push for effective reform of the UN system and global governance more generally — PICSOs may be well or better placed to do this since through political engagement, activists can indeed make some scenarios more likely — and other undesirable ones less likely and ultimately make more resolutions binding to member states.
- The risk of inaction in this realm is for new UN resolutions to only tinker with pat solutionsso that, by the end of the Sustainable Development Goals (2030), we will be again discussing these same issues.

Moreover, all the signing of letters of complaint and the writing of declarations and petitions, as well as the three-minutes-reading-of-statements at UN meetings PICSOs are allowed to make may make us feel better, but how much do they help? Do we follow-up on them?

 Finally here, I want to emphasize that nothing is going to come from the ‘member states or this or that UN agency or the international community *should*’ parlance in recommendations. World Bank Reports are full of these ‘shoulds’(!) and look where that has taken us. Assessing claim holders’ capacity and space to de-facto demand is thus part of the broader challenge. In short, any call must be coupled with human rights learning at the bases so as to help claim holders empower themselves to start demanding the needed changes. Otherwise, our calls will become yet more wish letters to Santa Claus that only bring us toys …‘batteries not included.’ Worldwide coordination among all social movements that support the human rights-based framework is thus the crucial challenge: forget relying on the ill-defined ‘international community!’

## What I Think Needs, Among Other, to Be Done

 [Actions suggested here to address the deplorable current situation in global governance are, again, brief and not exhaustive; they are presented in no particular order of priority and I am not as pretentious as to think I have the package-of-actions-to-follow to propose to you — they are rather terribly prescriptive and normative; they complement Labonté and colleagues’ and Meurs and colleagues’ views].

 The main challenges I suggest be addressed can be gathered under two rubrics:

## Need for Collective Action

 A strong advocacy work at UN agencies is needed in several fronts: First, I would say is to keep demanding resolutions do not require being passed by consensus, ie, allowing for member states voting for them thus eventually allowing minority reports. Second, is to be careful not to compromise when, so often — in a mockery of opening up to democratic decision-making — PICSOs are asked to comment on and/or endorse ‘zero’ or advanced drafts of official UN documents. This goes together with not accepting more promises in these documents if they do not go with concrete measures that can be legally enforced and monitored. This, I strongly feel is why so many resolutions end up with what only appears-to-be well hammered-out recommendations; in the end, the latter are only aspirations; without a commensurate call for matching policies based on legally enforceable measures, these resolutions are of no relevance to the fulfilment of “We The People of the United Nations” rights. Add to this the deceiving, poorly defined language used in these resolutions: no more stakeholders, no more loosely defined partnerships among unequal allies, no more non-state actors, no more evidence-based, no more international community, no more mutual accountability…

 To make progress on the above, the PICSOs communications capacity has to significantly increase and become more punching; since the traditional media are controlled by the forces of the status-quo, social media are the best option they have (I note that Twitter storms have achieved some victories, if limited).

 As a take-home message here for actions needed, consider: As much as more political analyses are needed, so are more political actions. I would therefore posit that to make sense of current world problems, we too often fall back on a ‘shish-kebab mentality.’ This much easier and convenient approach looks at the various problems affecting the world as if they were all separate events skewed together by tragedy or destiny. So, we set out to tackle each individual morsel …when the problem is in the skewer, ie, in the structural determinants or, if you wish, the common systemic drivers of the problems behind each morsel. These are linked to the prevailing neoliberal system that is at the very core-of and affecting each of the morsels. The point thus is: The focus has to be on changing the skewer as a means to more radically change the morsels. So, the morsels have to come together as a collective rather than letting themselves be pinched up individually on the skewer.

## Closer Zeroing in on Structural Determinants^[1]^

 PICSOs alone will hardly achieve the needed structural changes; this means they have to actively work with sympathetic UN member states willing to speak up in international fora partnering with PICSOs — since civil society representatives are not given the floor to openly demand the changes their respective constituencies call for. As important, is for them to connect and exchange analyses and tactics with like-minded social movements constituencies and other civil society platforms in an effort to broaden the mobilization around the structural determinants depicted in the shish-kebab above, from local to global levels. The rationale is that the broader the base of organized claim holders that can be reached to exercise counter-power, the more sustainable the outcome will be, noting that, for this to happen, claim holders must progressively get inside spaces where they have been traditionally uninvited and/or excluded. Some political parties and ‘sympathetic champions’ inside UN agencies and other international agencies ought not to be off-limits in this effort either since such persons do exist and are key assets and need to be nurtured and encouraged to speak out [A caveat here is to watch out for Business interest non-governmental organizations that pretend to be on the public interest’s side, but are hiding who their financial sponsors are. The tactic has been called ‘astroturfing’^4^].

 As a take-home message here for zeroing in on what is urgent, consider: Many small struggles are to coalesce; among other, this means additionally engaging with academics, trade unions and with youth and women’s and indigenous peoples grassroots organizations — emulating the climate movement and their effective denunciation, eg, the Fridays For Future movement and Greta’s Blah! Blah! Blah! Denunciation. This broadening of alliances is to include engagement with the different UN mandate holders (including UN special rapporteurs), as well as with the progressive organizations advocating for the struggle of PICSOs’ struggle in Geneva (the South Center, the Third World Network, the Europe-Third World Centre, Centre Europe-Tier Monde…), in the Netherlands Transnational Institute, Transnational Institute and in so many other places that I feel guilty not to mention.

 Last but not least, if PICSOs are to achieve much of the above, their internal organization must be strengthened so they can redouble their efforts to get involved, as well as to mentor more able spokespersons, especially young activists, to speak out.

## Bottom Line

 I started asking: Can we work towards building a people’s governance grounded in multilateralism and human rights? The answer may be to adopt new, more drastic and far-reaching ways of engagement. If this fails, PICSOs and people’s movements may as well ponder the alternative to leave working with UN bodies coopted by powerful interests and moving their demands to encourage action at grassroots organizations with a greater potential to influence governance decisions that break away from the neoliberal chokehold. I recognize we are not there yet. The example of the People’s Health Movement’s ‘WHO Watch’ active in the World Health Assembly and its Executive Board meetings every year adds an important action point suggestion here.^5^

 PICSOs and social movements are not giving priority to this grassroots mobilization yet. They are giving priority to continue staying in UN spaces to be watchdogs and to continue demanding conditions and procedures they want to see in place. In that sense, it is about resisting, but hardly about being radically forward-looking.

## An Afterthought

 In grieving for the alleged failures of our progressive struggles of the past, do weigh what may have happened if as PICSOs and social movements, we would not have engaged in those struggles!

## On a More Facetious Note

 En un café de Madrid escuché esta conversación, que mostraba un gran pesimismo, pero ningún dramatismo:

 Uno de los contertulios le decía a otro:

 -A mi, lo que más me gusta es perder a las barajas.

 -¿Pero es que no te gusta ganar?

 -¡Coño! ¿se puede? (you can https://deepl.com translate this).

## Acknowledgements

 Some concepts were inspired from the Civil Society Mechanism and Indigenous Peoples’ document ‘Towards a Strategy on Global Food Governance’ (https://www.csm4cfs.org/policy-working-groups/global-food-governance/). Otherwise, I acknowledge the generous inputs of Nora Mckeon, Sofia Monsalve, Raffaele Morgantini, Patti Rundall, Ted Greiner, David Legge, and Stefano Prato.

## Ethical issues

 Not applicable.

## Competing interests

 Author declares that he has no competing interests.

## Disclaimers

 The views expressed in this commentary are those of the author and do not constitute a position of the People’s Health Movement. The commentary is primarily, but not only, for reflection by colleagues and fellow travelers who, with me and for many years, have been quixotically fighting the windmills of global development governance.

## Endnotes

 [1] here I focus on challenges PICSOs ought to be picking up on.
